# Bacteria and Methanogens Differ along the Gastrointestinal Tract of Chinese Roe Deer (*Capreolus pygargus*)

**DOI:** 10.1371/journal.pone.0114513

**Published:** 2014-12-09

**Authors:** Zhipeng Li, Zhigang Zhang, Chao Xu, Jingbo Zhao, Hanlu Liu, Zhongyuan Fan, Fuhe Yang, André-Denis G. Wright, Guangyu Li

**Affiliations:** 1 Jilin Provincial Key Laboratory for Molecular Biology of Special Economic Animals, Institute of Special Animal and Plant Sciences, Chinese Academy of Agricultural Sciences, Changchun, China; 2 State Key Laboratory of Genetic Resources and Evolution, Kunming Institute of Zoology, Chinese Academy of Sciences, Kunming, China; 3 School of Animal and Comparative Biomedical Sciences, University of Arizona, Tucson, Arizona, United States of America; Free University of Bozen/Bolzano, Italy

## Abstract

The current study provides the insight into the bacteria in the gastrointestinal tract (GIT) and methanogens presented in the rumen and cecum of the Chinese roe deer (*Capreolus pygargus*). The ruminal, ileal, cecal, and colonic contents, as well as feces, were obtained from each of the three, free-range, roe deer ingesting natural pasture after euthanasia. For the bacterial community, a total of 697,031 high-quality 16S rRNA gene sequences were generated using high-throughput sequencing, and assigned to 2,223 core operational taxonomic units (OTUs) (12 bacterial phyla and 87 genera). The phyla Firmicutes (51.2%) and Bacteroidetes (39.4%) were the dominant bacteria in the GIT of roe deer. However, the bacterial community in the rumen was significantly (*P*<0.01) different from the other sampled regions along the GIT. Secondly, *Prevotella* spp., *Anaerovibrio* spp., and unidentified bacteria within the families Veillonellaceae and Paraprevotellaceae were more abundant in the rumen than in the other regions. Unidentified bacteria within the family Enterobacteriaceae, *Succinivibrio* spp., and *Desulfovibrio* spp. were more predominant in the colon than in other regions. Unidentified bacteria within the family Ruminococcaceae, and *Bacteroides* spp. were more prevalent in the ileum, cecum and fecal pellets. For methanogens in the rumen and cecum, a total of 375,647 high quality 16S rRNA gene sequences were obtained and assigned to 113 core OTUs. *Methanobrevibacter millerae* was the dominant species accounting for 77.3±7.4 (S.E) % and 68.9±4.4 (S.E) % of total sequences in the rumen and cecum of roe deer, respectively. However, the abundance of *Methanobrevibacter smithii* was higher in the rumen than in the cecum (*P* = 0.004). These results revealed that there was intra variation in the bacterial community composition across the GIT of roe deer, and also showed that the methanogen community in the rumen differed from that in the cecum.

## Introduction

The rumen is inhabited by a dense and diverse consortium of microorganisms, including bacteria, archaea, protozoa and fungi that have a symbiotic relationship with the ruminant, with bacteria playing the critical role in biomass degradation [1]. This has led to a variety of studies investigating rumen bacterial structure in domesticated ruminants, such as cows, sheep, yak, reindeer and sika deer [2]–[6]. It is well known that the rumen bacterial communities are significantly affected by the diet and ruminant species [7]–[10]. The diets of domesticated ruminants usually is comprised of high-quality forages or concentrates, whereas the diets of wild ruminants depend on the nature of the browse and forage available for ingestion at a given point in time within the environment.

Roe deer (*Capreolus pygargus*), a member of the family Cervidae, feeds mainly on grass, leaves, berries and young shoots, particularly very young, tender grass with a high moisture content. Given the difference of feeding strategies between wild and domesticated ruminants, one would expect that the bacterial populations in the rumen of roe deer should be distinct. To our knowledge, no published studies exist on the GIT bacterial composition of roe deer. Therefore, understanding the structure of the bacterial communities in the GIT of roe deer could improve our understanding of the breadth of microbial diversity in wild ruminants, and may be useful for developing new livestock management technologies, particularly in nutrition and sustain ability systems.

Enteric methane is a natural byproduct arising from microbial fermentation of feeds within the rumen and, to some extent, in the cecum [11], which is produced by methanogenic archaea (i.e., methanogens) utilizing hydrogen to reduce carbon dioxide to methane. Enteric methane not only contributes to global warming and climate change, but also represents a significant energy loss to cattle ranging from 2% to 12% of gross energy intake [12], [13]. Decreasing methane emissions from livestock have important environmental and economic implications. Notably, methane production of roe deer is relatively lower than other ruminants [14]. Therefore, examining the methanogens in the rumen and cecum of roe deer will help us to better understand methanogen ecology, and may be useful in developing strategies to decrease enteric methane emissions.

Here, the current study performed the high-throughput sequencing based on the 16S rRNA gene in order to: (i) examine and compare the bacterial community composition in the GIT of roe deer; and (ii) investigate the methanogen community in the rumen and cecum of roe deer.

## Materials and Methods

### Animals and sampling

Three healthy, two year old, male roe deer (*Capreolus pygargus*), about 25 kg, were used in this study. The free ranging animals were reared by local farmer grazing pasture and maintained in local mountains of Chifeng City, Inner Mongolia Autonomous Region in China. The animals were euthanized by intravenous injection of barbituric acid (90 mg/kg body weight) before the morning feeding. The protocol was approved and authorized by the Chinese Academy of Agricultural Sciences Animal Care and Use Committee. There were no specific permissions required for this animal study.

Five areas (top, medium, bottom, left and right) of rumen contents, including solid and liquid fractions, were separately sampled. The luminal contents including ileum, cecum and colon, were also collected. The ileum luminal contents were obtained from the beginning, middle and end sections. For the cecum, contents from the top, medium and bottom sections were separately collected. Luminal contents in the internal and external handles of the ascending colon, the transverse colon, and the descending colon were also sampled. Feces were also taken from the terminal part of the rectum. After the sampling, all samples were immediately frozen in liquid nitrogen and then stored at −80°C until further analysis.

### DNA extraction

Total genomic DNA was extracted from the samples of each animal using a QIAamp DNA Stool Mini Kit (QIAGEN, Valencia, CA) according to the manufacturer's instructions. Genomic DNA from different location (see above description in sampling) within the same regions of the GIT (rumen, ileum, cecum, colon and fecal pellets, respectively) were pooled together in equivalent amounts, and then PCR amplified.

### Amplification of target genes and high-throughput sequencing

The V3–V4 region of the bacterial 16S rRNA gene was amplified using primers 338F (5′-ACTCCTACGGGAGGCAGCA-3′) and 806R (5′- GGACTACHVGGGTWTCTAAT -3′) for all samples [15]. The variable region of 16S rRNA gene from methanogens was amplified using primers 519F (5′-CAGCMGCCGCGGTAA-3′) and 976R (5′-CCGGCGTTGAMTCCAATT-3′) for the rumen and cecum samples [16], [17]. Each primer was designed that contained: 1) the appropriate Illumina adapter sequence allowing amplicons to bind to the flow cell; 2) an 8 bp index (i.e., barcode) sequence; and 3) gene-specific primer sequences as described above. Resulting amplicons were purified using QIAquick PCR Purification Kit (QIAGEN, Valencia, CA). The purified amplicons were then pooled in equimolar concentrations, and the amplicon libraries were quantified using QuantiFluor-P Fluorometer (Promega, CA). PhiX Control library (Illumina) was combined with the amplicon library (expected at 20%), and then sequenced on the MiSeq platform.

### Bioinformatic analysis

The read pairs were extracted and concatenated according to the barcodes for each paired read from each sample generating contigs. Contigs with an average quality <20 over a 10 bp sliding window were also culled. The retained contigs were processed and analyzed using QIIME 1.7.0 following the pipeline described by Caporaso et al. [18]. Contigs were examined for quality control using the following criteria: the minimum sequence length was 400 nt; the maximum sequence length was 500 nt; minimum quality score was 25; the maximum number of errors in the barcode was 0; the maximum length of homopolymer run was 6; the number of mismatches in the primer was 0; ambiguous and unassigned characters were excluded. The remaining sequence was clustered into operational taxonomic units (OTUs) using Usearch61 according to the sequence identity of 97% at species level [19]. Representative sequences of OTUs were aligned to the Greengenes database for bacteria and methanogens 16S rRNA genes [20]. Potential chimera sequences were removed using Chimera Slayer [21]. The remaining representative OTUs were screened using Basic Local Alignment Search Tool with default parameters by QIIME 1.7 [22]. The OTU count table was constructed basing on the indentified OTUs and corresponding taxonomies. The OTUs that were found in ≥50% samples were retained for the further analysis. Alpha-diversity from all samples including Shannon-Wiener, Simpson, and Chao 1 indices were also calculated from QIIME 1.7.0 [18]. The variations of bacterial communities of all samples from the three animals were visualized based upon the beta-diversity, principal coordinate component analysis. Hierarchical clustering basing the genus-level relative abundance in all samples was used to perform the heatmap. Sequences from the present study were deposited to the NCBI Sequence Read Archive under the accession number SRP045434.

### Statistical analysis

Statistical analysis was performed using the SigmaPlot 12.0 (Systat Software, Inc.). Variations among the GIT (rumen, ileum, cecum, colon and fecal pellets) were checked for normal distribution using the Shapiro-Wilk test. When normally distributed, multiple samples comparisons were performed using one-way analysis of variance (ANOVA) (parametric), and using Kruskal-Wallis one-way ANOVA on ranks (non-parametric) for abnormal distribution with the significant value of *P*<0.05.

## Results

### Summary of the sequencing data

A total of 973,576 raw sequences were generated for the bacteria and 389,434 were generated for the methanogens. After filtering, quality control, chimera removal, and using a 97% sequence identify criterion, we indentified and characterized the core OTUs shared by more than half samples, that were deemed to be members of a core microbiota. The results showed that 697,031 high-quality bacterial sequences were assigned to 2,223 core OTUs ranging from 1,331 to 2,705 OTUs for each sample (Table 1). Moreover, 375,647 high-quality methanogen 16S rRNA gene sequences were assigned to 113 core OTUs, with the range of 102 and 111 OTUs for each sample (Table 1). By using the estimation of Good's Coverage [23], 99.1±0.1 (Mean ± standard error (S.E)% of the total bacterial species and 99.9±0.003% of the total methanogen species were represented in any given sample, ensuring the completeness of our data for the next set of analyses.

**Table 1:** Summary of next generation sequencing data.

M	Samples	Sequences	OTUs	Good's Coverage	Shannon	Simpson	Chao 1
Bac	Rumen_1	41215	1331	0.99	7.26	0.98	1928.51
Bac	Rumen_2	44350	1508	0.99	7.29	0.97	2057.81
Bac	Rumen_3	39766	1421	0.99	7.47	0.99	1945.74
Bac	Colon_1	62837	1812	0.99	8.19	0.99	2127.83
Bac	Colon_2	48607	1851	0.99	7.50	0.96	2117.59
Bac	Colon_3	65899	2004	0.99	8.07	0.98	2253.62
Bac	Ileum_1	39751	1993	0.99	8.53	0.99	2201.22
Bac	Ileum_2	55079	2075	1.00	8.46	0.99	2193.45
Bac	Ileum_3	27464	1852	0.98	8.31	0.99	2209.05
Bac	Cecum_1	32116	1937	0.99	8.55	0.99	2246.11
Bac	Cecum_2	62533	2072	1.00	8.32	0.99	2246.94
Bac	Cecum_3	53603	2037	1.00	8.43	0.99	2151.85
Bac	Feces_1	29532	1861	0.98	8.44	0.99	2217.72
Bac	Feces_2	45850	2048	0.99	8.24	0.98	2229.94
Bac	Feces_3	48429	2005	0.99	8.44	0.99	2224.95
Met	Rumen_1	69329	109	1.00	1.96	0.57	121.75
Met	Rumen_2	60328	102	1.00	0.84	0.20	122.50
Met	Rumen_3	56529	103	1.00	1.50	0.43	115.71
Met	Cecum_1	53951	109	1.00	1.69	0.46	138.30
Met	Cecum_2	68931	108	1.00	1.60	0.50	129.00
Met	Cecum_3	66579	111	1.00	2.21	0.63	129.00

M, Microorganisms; Bac, Bacteria; Met, Methanogen; OTUs, operational taxonomic units.

### Bacterial communities in the GIT of roe deer

**Figure 1:**
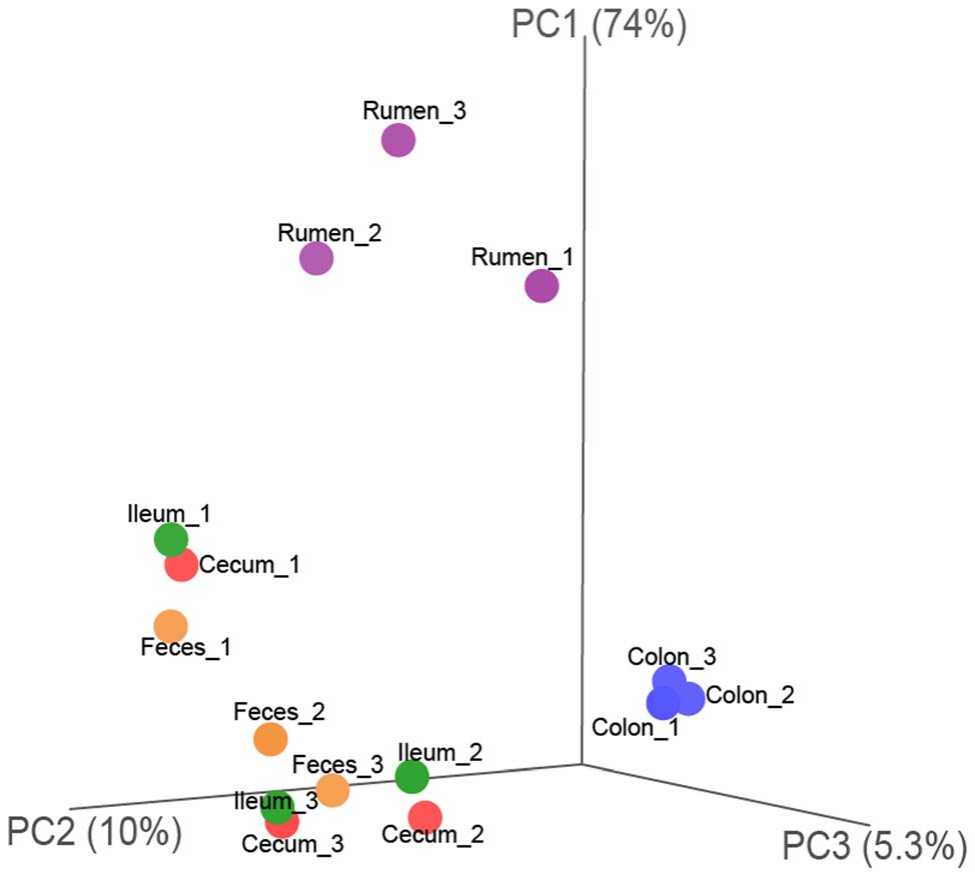
16S rRNA gene surveys reveal hierarchical partitioning of all 15 samples. Bacterial communities were clustered using principal coordinate analysis of the full-tree-based Unifrac matrix. Each point corresponds to a sample colored to indicate locations in the GIT. Three principal components (PC1, PC2, and PC3) explained 89.3% variation.

To investigate variations of bacterial distribution among the GIT of roe deer, the bacterial diversity in the GIT was compared. Species richness as estimated by Chao1 index in the colon (*P* = 0.009), ileum (*P* = 0.003), cecum (*P* = 0.002) and feces (*P* = 0.002) was higher than that in the rumen, and species diversity through Shannon index was found to be higher in the colon (*P* = 0.029), and in the ileum (*P*<0.001), cecum (*P*<0.001) and feces (*P*<0.001) compared to that in the rumen (Fig. S1). Additionally, comparison of bacterial communities based on all core OTUs by principal coordinate analysis showed that the bacterial communities in the rumen and colon were different from each other, and from the bacterial communities in the ileum, colon and fecal pellets, while the bacterial communities were similar within the ileum, colon, and in fecal pellets (Fig. 1).

### Bacterial community composition in the GIT of roe deer

**Figure 2:**
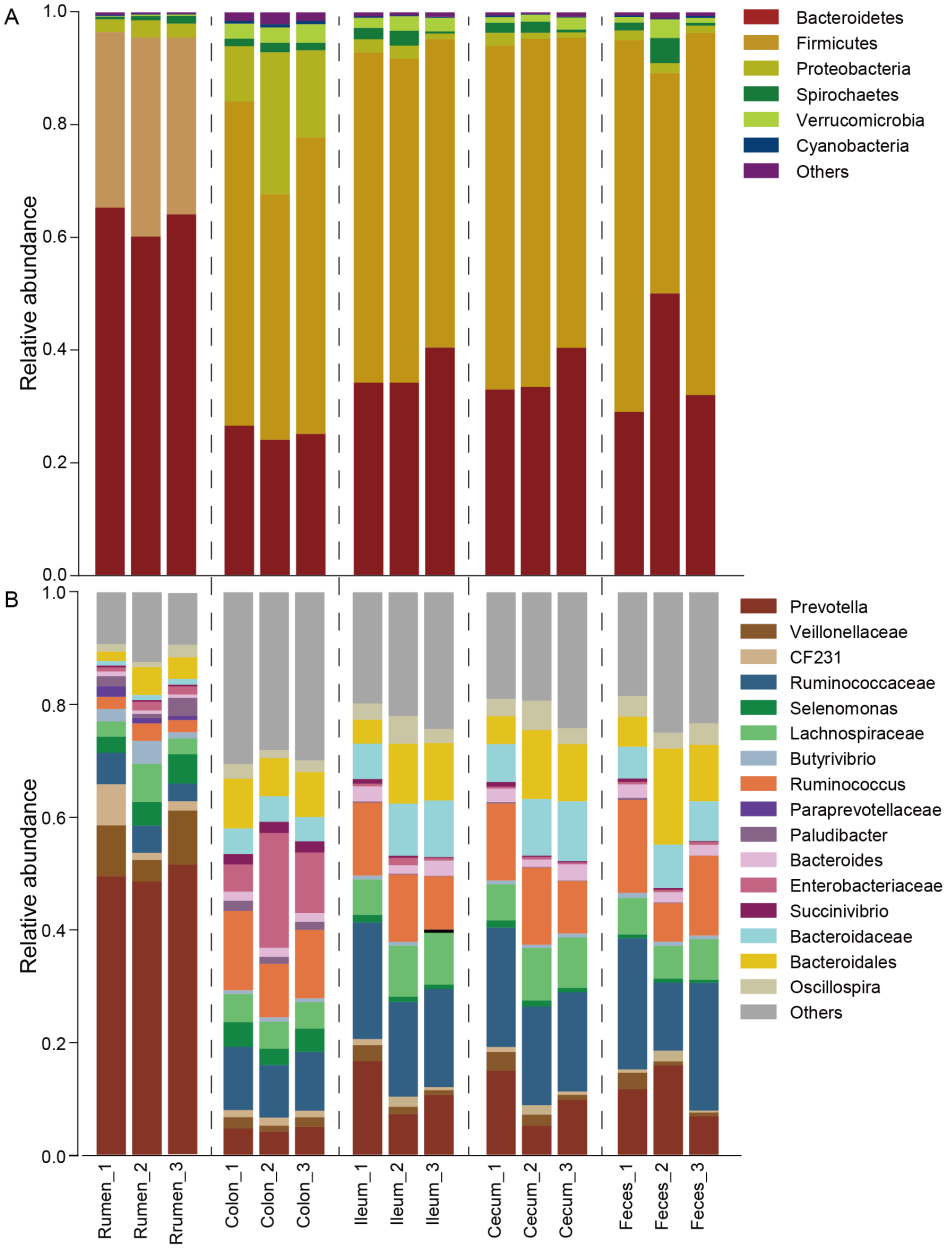
The relative abundance of bacterial communities at phylum (A) and genus (B) levels in the GIT of three roe deer.

**Figure 3:**
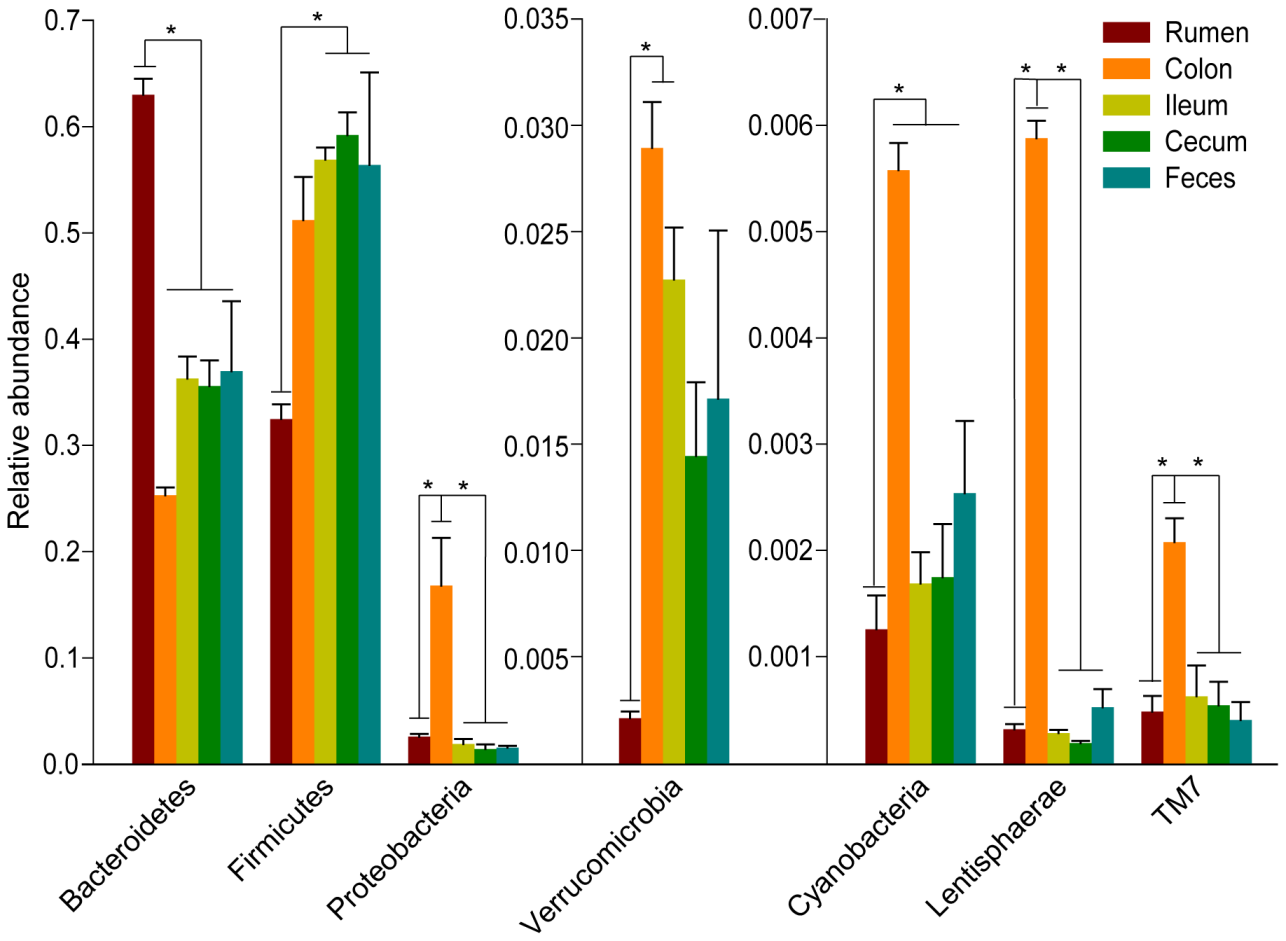
Bacterial phyla with significant differences in the GIT of three roe deer. The asterisk means the significance at *P*<0.05.

Taxonomic assignment based on the core OTUs at the phylum level showed that a total of 12 bacterial phyla were identified within the GIT of roe deer. The phyla Firmicutes (rumen: 32.5±1.4%; colon: 51.2±4.1%; ileum: 56.9±1.1%; cecum: 59.2±2.1%; feces: 56.4±8.7%) and Bacteroidetes (rumen: 63.0±1.5%; colon: 25.3±0.7%; ileum: 36.3±2.1%; cecum: 35.6±2.4%; feces: 37.0±6.6%) were the dominant bacteria. Other phyla were also present but at lower percentage (Fig. 2A). Examining each sample composition at the phylum level also revealed the noticeable differences between individual animals (Fig. 2A). Moreover, there were significant variations of bacterial composition at phylum level in the GIT of roe deer. Bacteria belonging to the phylum Bacteroidetes (63.0±1.5%) were more predominant in the rumen than other regions (*P*<0.0001), while the proportion of Firmicutes (32.5±1.4%) was much higher in the ileum, cecum and feces than in the rumen (*P*<0.005) (Fig. 3). The relative abundance of bacteria belonging to the phylum Proteobacteriawere more prevalent in the colon (16.8±4.4%) than in the other regions along the GIT (*P*<0.001) (Fig. 3). In addition, bacteria belonging to the phyla Verrucomicrobia, Cyanobacteria, Lentisphaerae and TM7 also differed along the GIT (*P*<0.005, Fig. 3).

We next performed the analysis of the composition and abundance of the core bacterial community based on the core OTUs at genus level, and indentified a total of 87 genera throughout the GIT of roe deer (Fig. 2B). In the rumen, *Prevotella* (49.9±0.7%), unidentified bacteria within the families Veillonellaceae (7.5±1.8%) and Paraprevotellaceae (1.2±0.3%) were the top three genera. In the colon, unidentified bacteria within the family Enterobacteriaceae (12.0±4.5%), *Ruminococcus* spp. (11.9±1.3%), and unidentified bacteria within the family Ruminococcaceae (10.3±0.5%) were the dominant bacteria. And in the ileum, cecum and feces, unidentified bacteria within the family Ruminococcaceae (ileum: 18.3±1.2%; cecum: 18.7±2.1%; feces: 19.3±6.3%), *Prevotella* spp. (ileum: 11.6±2.7%; cecum: 10.0±2.8%; feces: 11.6±2.6%) and *Ruminococcus* spp. (ileum: 11.5±1.0%; cecum: 12.2±1.5%; feces: 12.5±2.9%) were the predominant bacteria.

**Figure 4:**
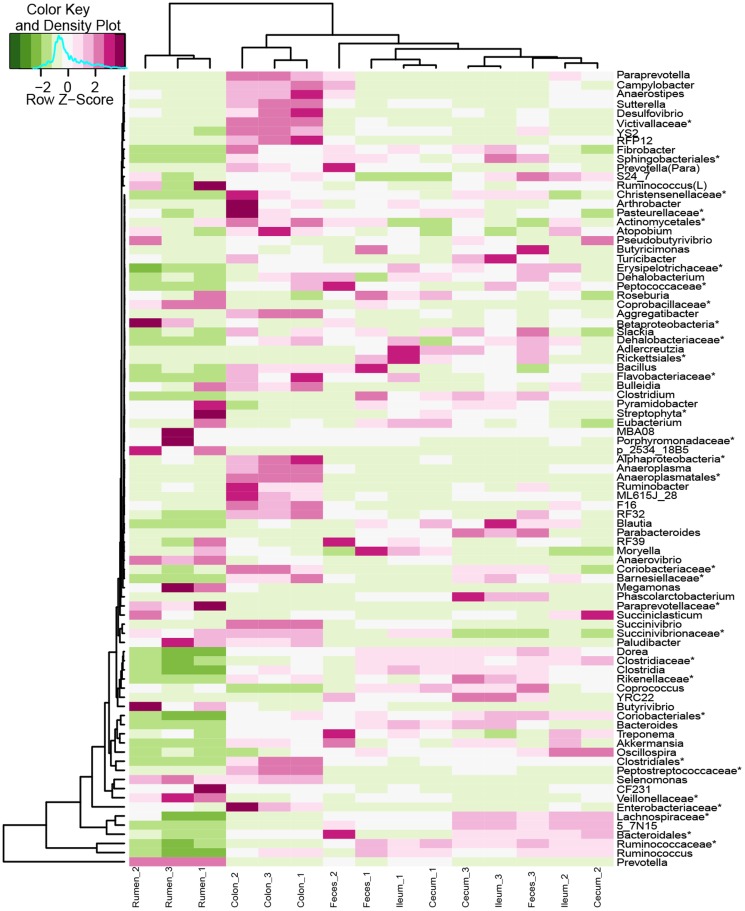
Heatmap analysis showing the distributions of bacterial communities at genus level in the GIT of three roe deer. Individual cells are color-coded according to raw Z-scores to show the abundance of a particular genus in each region. The asterisk means the unclassified bacteria at the family, order, or class levels.

However, when the composition of the bacterial communities across the different GIT regions was evaluated using heatmap analysis, the study observed a dissimilarity in bacterial composition at the genus level in the GIT of roe deer. The rumen bacterial communities clustered separately from ileum, cecum, colon and fecal pellets. In other samples, the bacterial communities in the ileum, cecum, and in fecal pellets clustered more closely to each other, and away from the colonic bacterial community (Fig. 4).

**Figure 5:**
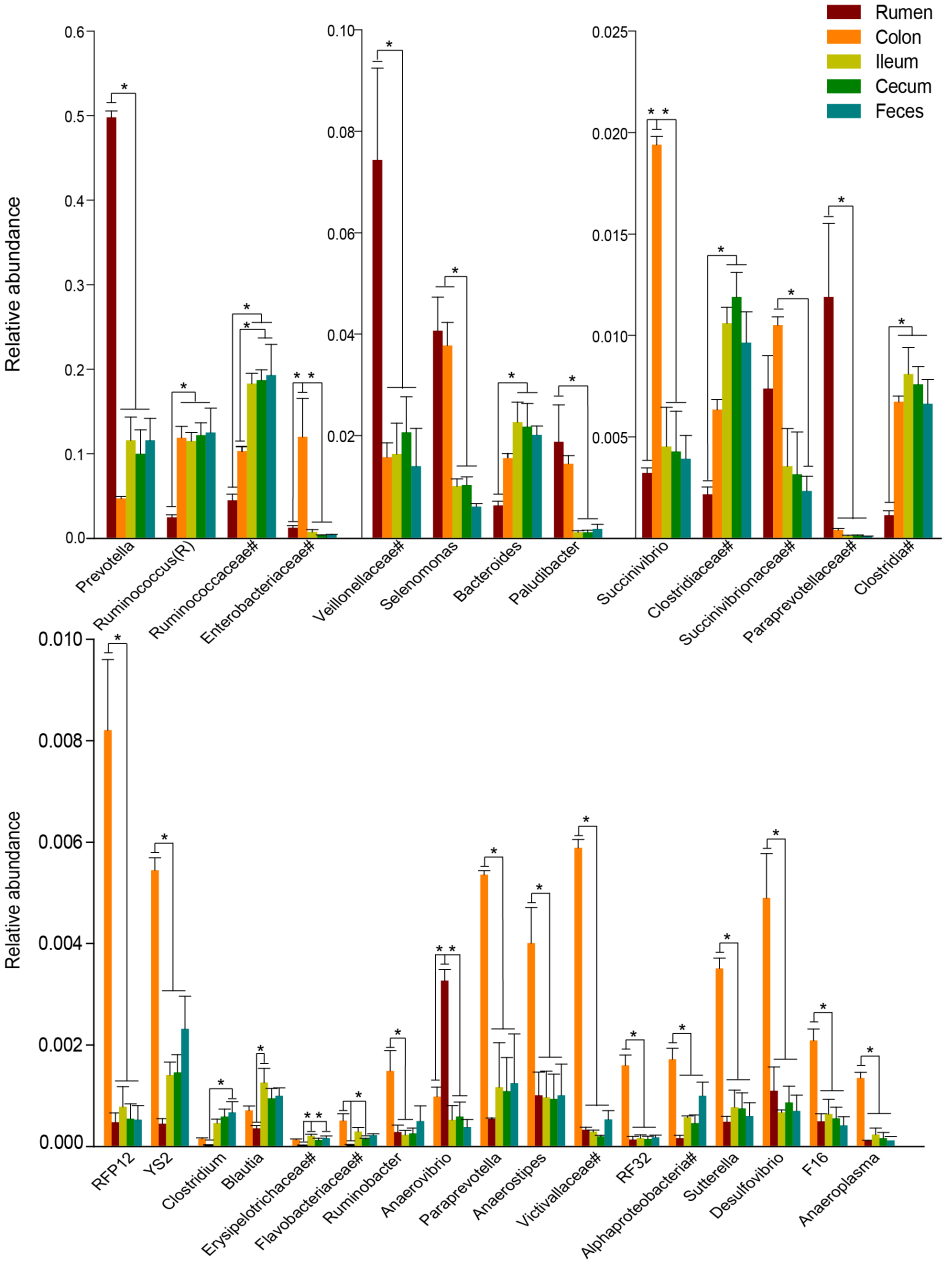
Significant difference at genus level of bacterial communities along the GIT of three roe deer. The asterisk indicates *P*<0.05, and # means the unclassified bacteria at the family, order, or class levels.

In further, we compared the relative abundance of all genera in the GIT. Most of the core bacterial community varied in abundance across the regions along the GIT (Fig. 5). *Prevotella* spp. (49.9±0.7%, *P*<0.001), *Anaerovibrio* spp. (0.3±0.02%, *P*<0.001), unidentified bacteria within the families Veillonellaceae (7.5±1.8%, *P*<0.005), and Paraprevotellaceae (1.2±0.3%, *P* = 0.005) were more abundant in the rumen than in other regions, while the distribution of *Ruminococcus* spp. (2.5±0.3%, *P*<0.005) was significantly lower in the rumen than in other regions. Additionally, the relative abundance of *Paludibacter* spp. (1.9±0.7%, *P*<0.05) and *Selenomonas* spp. (4.1±0.7%, *P* = 0.001) in the rumen was higher than in the ileum, cecum, and feces (Fig. 5). In the colon, unidentified bacteria within the family Enterobacteriaceae (12.0±4.5%, *P*<0.05), as well as *Succinivibrio* spp. (1.94±0.04%, *P*<0.001), *Paraprevotella* spp. (0.54±0.008%, *P*<0.01), *Desulfovibrio* spp. (0.49±0.09%, *P*≤0.001), *Anaerostipes* spp. (0.4±0.07%, *P*<0.05), *Sutterella* spp. (0.35±0.02%, *P*<0.001) and *Anaeroplasma* spp. (0.14±0.01%, *P*<0.001) were more abundant in the colon than in other regions of the GIT (Fig. 5). Furthermore, in the ileum, cecum and fecal pellets, unidentified bacteria within the family Ruminococcaceae (ileum: 18.3±1.2%, cecum: 18.7±2.1%, fecal pellets: 19.3±6.3%) was more abundant than in rumen (4.5±2.1%, *P*≤0.001), and the relative abundance of *Bacteroides* spp. (ileum: 2.3±0.3%, cecum: 2.2±0.4%, fecal pellets: 2.1±0.1%) was higher than in the rumen (0.6±0.08%, *P*<0.05) (Fig. 5).

### Methanogen communities and composition in the rumen and cecum of roe deer

**Figure 6:**
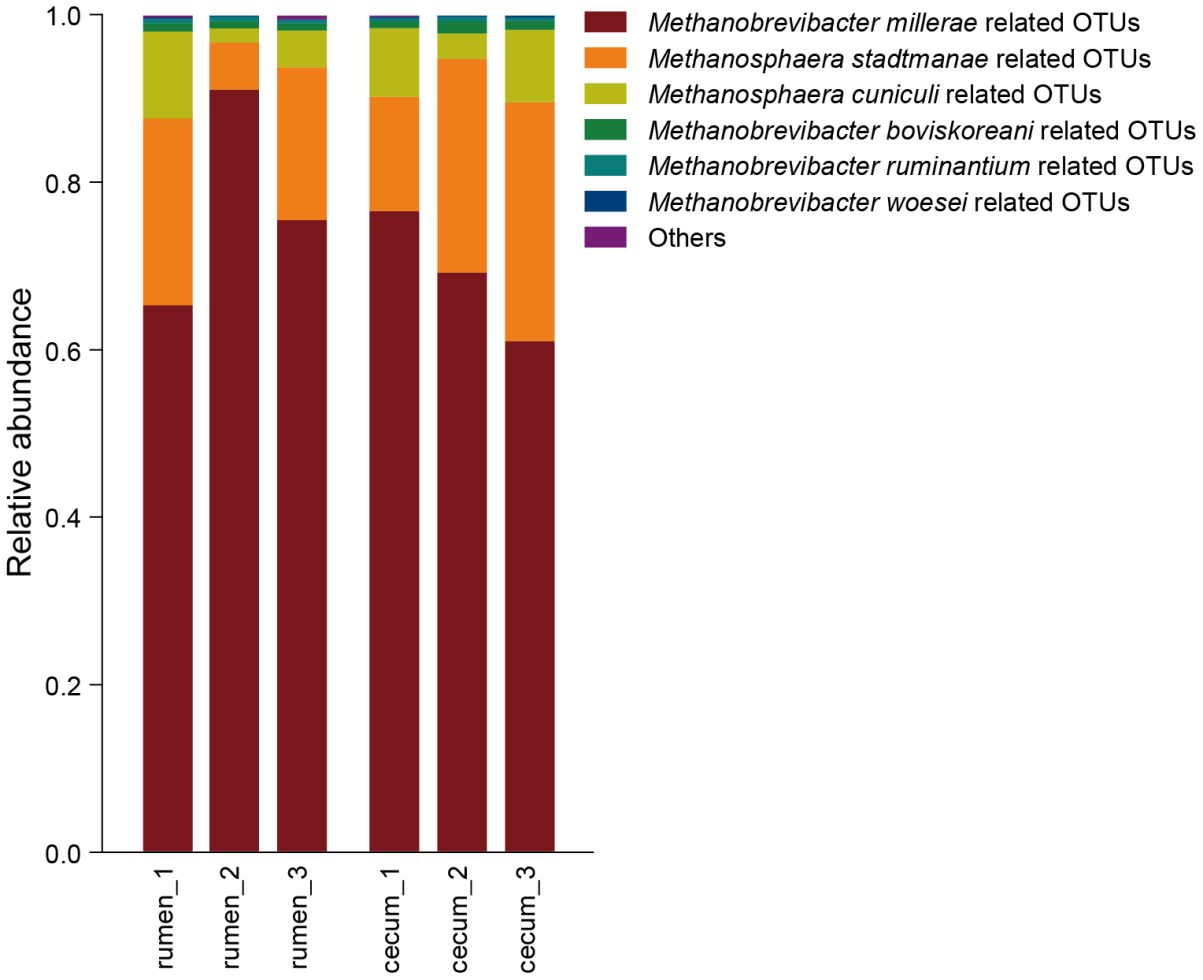
The relative abundance of methanogen in the rumen and cecum of three roe deer.

**Figure 7:**
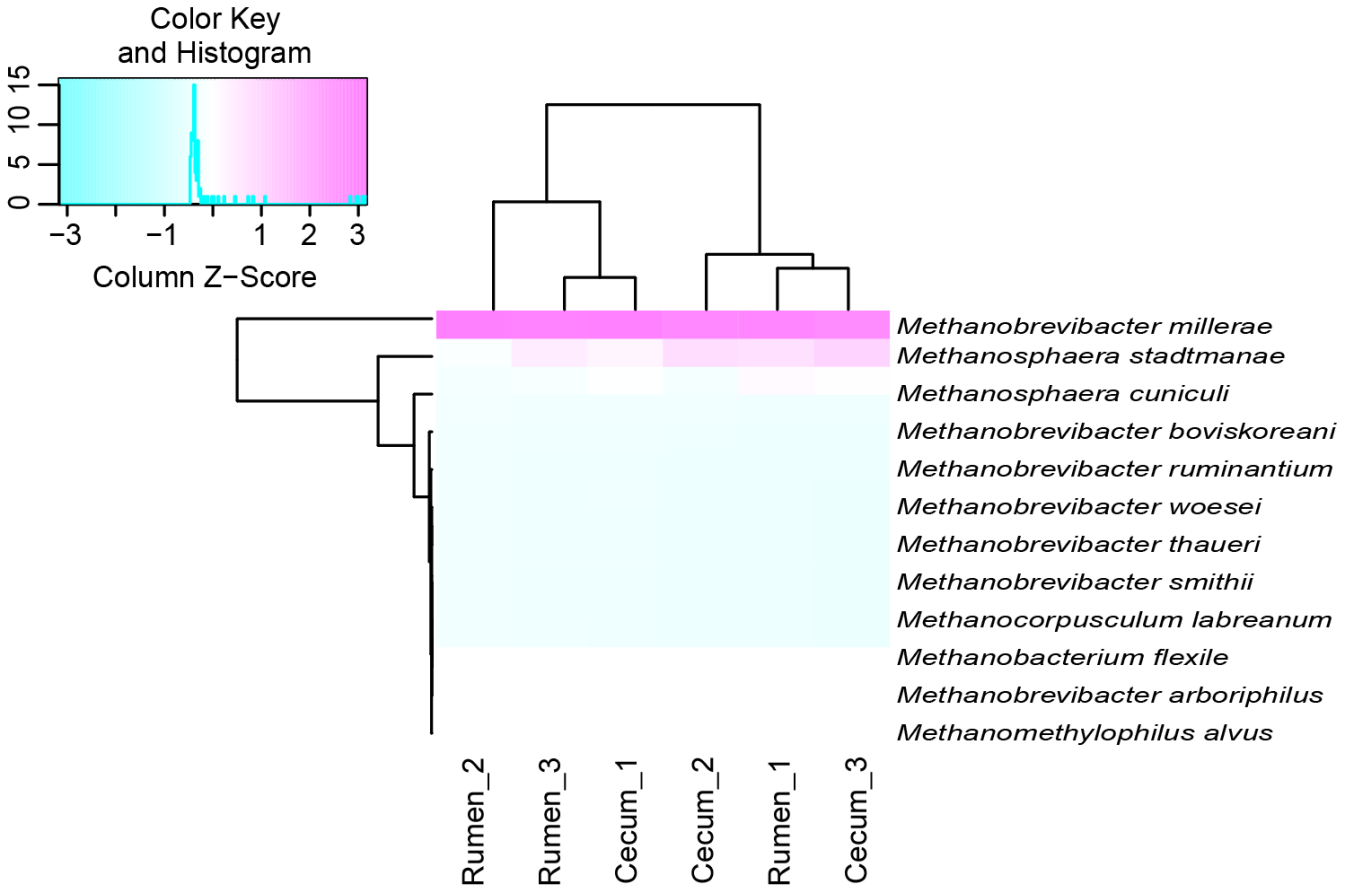
Heatmap analysis showing the distributions of methanogens communities in the rumen and cecum of three roe deer. OTU, operational taxonomic units.

The Chao 1 index of methanogens in the cecum (132.1±3.1) was higher than in the rumen (119.9±2.1) of roe deer (*P* = 0.03). To examine the methanogen composition in detail, these representative sequences from each OTU were entered into GenBank's Nucleotide Basic Local Alignment Search Tool [24], and the nearest valid species was identified along with the percent sequence similarity (Table S1). Similar to the results of bacteria, the methanogens composition was also varied between individuals. As shown in Fig. 6, *Methanobrevibacter millerae* was the dominant species accounting for 77.3±7.4% and 68.9±4.4% of total sequences in the rumen and cecum of roe deer, respectively. *Methanosphaera stadtmanae* was the second dominant species in the rumen and cecum represented by 8.7±5.0% and 7.9±4.5% of total sequences, respectively. Moreover, *Methanosphaera cuniculi* also accounted for 4.4±2.5% and 3.1±1.8% of total sequences in the rumen and cecum, respectively. However, the principal coordinate analysis revealed that cecum samples grouped more closely together than did the rumen samples (Fig. S2). Additionally, the heatmap analysis of methanogens also showed that the methanogens communities in the rumen and cecum were differed (Fig. 7). When the methanogens composition was compared using ANOVA, the result showed that the proportion of *Methanobrevibacter smithii* was higher in rumen (0.07±0.003%) than in the cecum (0.05±0.003%, *P* = 0.004).

## Discussion

An understanding of the microbial communities in the GIT was of great importance for the animal's performance. Therefore, the objective of the present study was to examine the bacterial community composition in the GIT and the methanogens community in the rumen and cecum of roe deer.

Analysis of the bacterial diversity (Shannon index) and richness (Chao 1 index) showed that the bacterial community in the rumen was significantly lower than that in the intestine and feces (Fig. 1, 4 and S1), indicating that the bacteria community composition varied across different GIT regions of roe deer. This is consistent with other studies investigating the variations of microbiota among the GIT regions of dairy cattle, steers, pre-weaned calves and moose [25]–[28]. This shift may correspond to the functional differences of the GIT (e.g. fermentation process) [29]. However, the bacterial diversity was higher in the rumen of steer and pre-weaned calves than that in the intestine and feces [27], [30]. This difference is likely explained by the physical or chemical parameter changes, or the unknown host factors selecting the bacterial community along the GIT of roe deer. Moreover, similar to the findings of Jami and Mizrahi [31], the present study showed that the bacteria and methanogens community were varied across individual animals, indicating that more animals should be included in future studies.

Taxonomic assignment based on the core OTUs showed that *Prevotella* spp. was conserved in the GIT, and was much more abundant in the rumen than in other regions. Similarly, *Prevotella* spp. was also the dominant bacteria in the rumen of elk, white tailed deer, sika deer, reindeer and moose [3], [6], [32], [33]. This may be the result of the co-evolution between the cervid host and their rumen bacterial community. Bacteria belonging to the genus *Prevotella* contain highly active hemicellulolytic and proteolytic enzymes [34], which can degrade non-cellulosic plant polysaccharides, starch, xylan, lignans and pectin [35], [36]. Additionally, *Prevotella* spp. comprised a large part of the genetic and metabolic diversity in rumen microbial communities [37], [38]. Metagenomic analyses also suggested that *Prevotella* spp. played a potential role in cellulose degradation in the foregut of the Tammar wallaby and in the rumen of Svalbard reindeer [39], [40]. Some studies found that the proportion of *Prevotella* spp. in the rumen was increased in higher concentrate diets [9], [41]. However, the diets of roe deer were comprised of pastures. Therefore, these results indicated that *Prevotella* spp. may play a key function in degrading the plant fibers in the rumen ecology of roe deer.

Furthermore, unidentified bacteria within the family Veillonellaceae were more prevalent in the rumen than in other regions. Similarly, de Menezes et al. [10] found that Veillonellaceae bacteria comprised up to 3% of all sequences in the rumen of cows fed pasture. Hooda et al. [42] found that the relative abundance of bacteria belonging to the family Veillonellaceae was increased when soluble corn fiber was part of an adult diet. Thus, these bacteria within the family Veillonellaceae may also play a critical role in the fermentation of plant fibers in the rumen of roe deer.

The results also showed that unidentified bacteria within the family Enterobacteriaceae, *Succinivibrio* spp. and *Desulfovibrio* spp. were more abundant in the colon compared to the other regions along the GIT. Ishaq and Wright [28] also found that Enterobacteriaceae bacteria were abundant in the colon of moose. *Desulfovibrio* spp. is an important sulfate-reducing bacterium in the rumen [43]. Arumugam et al. [44] speculated that *Desulfovibrio* spp. may enhance the rate-limiting mucin desulphation step of *Prevotella* spp. by removing the sulfate. Thus, *Desulfovibrio* spp. may have special roles in the colon of roe deer. Moreover, *Succinivibrio* spp. were found in high numbers in animals fed high-starch containing large amounts of rapidly fermentable carbohydrates, and involved in the digestion of starch in the rumen [45], [46]. The indigestible dietary substrates including cellulose and starch were fermented in the colon [47], [48]. Enrichment of these bacteria species in the colonic environments may be suggestive of a role in carbohydrate metabolism.

The present study found that the distribution of unidentified bacteria belonging to the family Ruminococcaceae, and *Bacteroides* spp. were more abundant in the ileum, cecum and fecal pellets than in the rumen. In contrast, Malmuthuge et al. [27] found that *Bacteroides* spp. and *Faecalibacterium* spp. were prevalent in the cecum, and *Lactobacillus* spp., *Clostridium* spp., and *Sharpea* spp. were abundant in the ileum of pre-weaned calves. Barker et al. [49] revealed that *Alistipes* spp., *Cloacibacillus* spp., and *Ruminococcus* spp. were prevalent in the cecum of the koala, and the predominant genera in the koala feces were *Bacteroide*s spp. and *Ruminococcus* spp. The dissimilarity may be due to the host genetics, age and dietary composition.

The bacterial community composition may contribute to the methanogen composition as the interactive relationships between rumen bacteria and methanogens were observed in previous study [50]. The current study revealed that *Methanobrevibacter* phylotypes were dominant in both the rumen and cecum of roe deer, consisting with the results of other ruminants studied world-wide [51]–[53]. At the species level, the rumen and cecum of roe deer were mostly dominated by *Mbr. millerae*. However, previous studies found that *Methanobrevibacter gottschalkii* was more abundant in the rumen of sheep from Venezuela [54], and in the foregut of wallabies from Australia during spring time [55], In addition, *Methanobrevibacter ruminantium* was higher in Holstein dairy cows fed different forages (USA and Canada) [56]–[58], and in corn-fed cattle from the province of Ontario (Canada) [59]. On the other hand, the dominant methanogens of roe deer were similar to the findings of the alpaca forestomach, which was mostly *Mbr. millerae* [60]. Similarly, alpacas produce less methane than cattle [61]. It is credible that the composition of the methanogen community may play more important roles in enteric methane production, rather than the density of methanogens [62]–[64]. Therefore, the discovered methanogen composition may be responsible for the low methane emission of roe deer [14]. However, the discovered methanogen communities in other regions of the GIT should also be noticed in further studies.

Interestingly, methanogens composition appears to be more similar within other cervid hosts. *Methanobrevibacter ruminantium*, *Mbr. gottschalkii* and *Methanosphaera* spp. were prevalent in the rumen of red deer in New Zealand [65]. The order Methanoplasmatales including the TALC/RCC methanogen lineages (Thermoplasmatales-related archaea) and Candidatus *Methanomethylophilus alvus*, *Methanomassiliicoccus luminyensis*, and *Mbr. millerae* were dominant in the rumen of Svalbard reindeer [66]–[69]. Similarly, *Mbr. millerae* was the most abundant methanogen in the rumen of sika deer fed corn stover and oak leaf based diets [70]. These results suggested that the host genetics may play a critical determinant in the diversity of methanogen.

The study further showed that the methanogen community in the cecum differed from that in the rumen. Similarly, Popova et al. [52] also found that the methanogens in rumen and cecum of wheat- and corn-fed lambs were significantly different. Moreover, the methanogen community in the cecum was more diverse than in the rumen, agreeing with the findings of Frey et al. [25], who also found the proliferation of methanogens in the ileum of cow as compared to the rumen. These results suggested that methanogens proliferated in the intestinal tract of roe deer, which may be related to the source and amount of the substrate in the cecum for methanogens, the existence of reductive acetogenesis, and the absence of hydrogen-producing protozoa in the cecum. In total, these findings indicate that the role of methanogens in both the rumen and cecum should be considered in future studies.

This study also found that *Mbr. smithii* was more prevalent in the rumen than in the cecum, and that *Mbr. millerae* tended to be more abundant in the rumen, while *Methanosphaera* spp. was prevalent in the cecum. Kittelmann et al. [50] found *Methanosphaera* spp. was negatively related to *Methanobrevibacter* spp. in the rumen, as these methanogens may compete for hydrogen for the production of methane [71], [72]. Moreover, these changes may be related to the alteration of substrate for methanogens, which was likely to arise from the changes of bacterial communities. For example, Ruminococcaceae bacteria were more abundant in the cecum than in the rumen, which produced large amounts of hydrogen in the degradation of cellulose [73]. On the other hand, *Methanobrevibacter* spp. had relatively higher hydrogen thresholds compared to species of *Methanosphaera* [74]. This could partially explain the different abundance of methanogens in the rumen and cecum. In future studies, the interactive relationships between the methanogen and bacterial communities warrant further investigation.

In conclusion, data presented here showed that there were intra variations in the bacterial communities across the GIT of roe deer. Increasing our knowledge of the bacterial communities among the GIT could help to improve the productivity of roe deer. Moreover, this study indicated that *Mbr. millerae* was the dominant methanogen in the rumen and cecum of roe deer, which could partially explain the low enteric methane emission of roe deer. The relationships between the predominant *Methanobrevibacter*, (e.g., *Mbr. millerae*) and methane output in the rumen of roe deer, warrant further investigation. Furthermore, the methanogen community in the rumen differed from that in the cecum of roe deer, suggesting that the impact of methanogens in both the rumen and cecum on methane emission should be considered in future studies.

## Supporting Information

Figure S1 **Comparison of the diversity indices of bacterial communities in the GIT of three roe deer.** The asterisk means *P*<0.05.
(TIF)

Figure S2 **16S rRNA gene surveys reveal hierarchical partitioning of all 6 samples.** Methanogens communities were clustered using principal coordinate analysis of the full-tree-based Unifrac matrix. Each point corresponds to a sample colored to indicate locations in the GIT. Three principal components (PC1, PC2, and PC3) explained 88.8% variation.
(TIF)

Table S1 **The results of Basic Local Alignment Search Tool for the representative sequences of methanogen OTUs.** OTU, operational taxonomic units.
(DOCX)
