# Supplementary figures and images for: Bacteria and Methanogens Differ along the Gastrointestinal Tract of Chinese Roe Deer (Capreolus pygargus)
Source: PLoS One. 2014 Dec 9;9(12):e114513. doi: 10.1371/journal.pone.0114513 (PMC4260832; doi:10.1371/journal.pone.0114513)

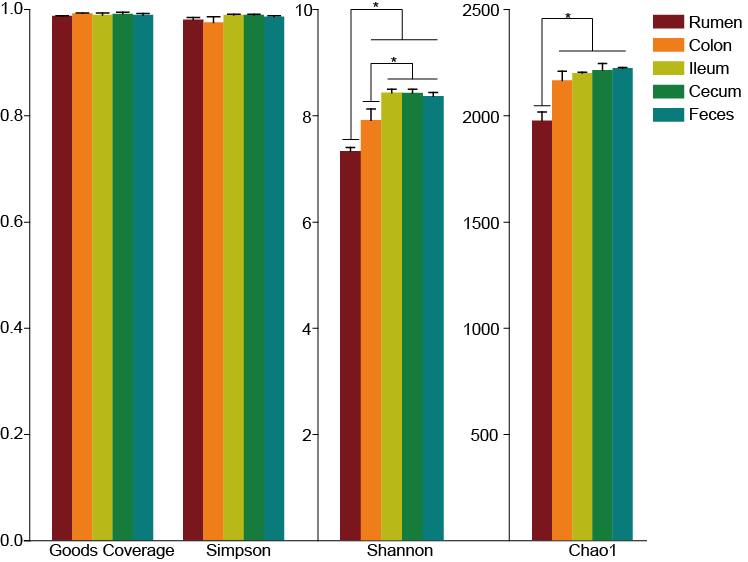

Supplement: Figure S1 — Comparison of the diversity indices of bacterial communities in the GIT of three roe deer. The asterisk means P<0.05. (TIF) [file pone.0114513.s001.tif]

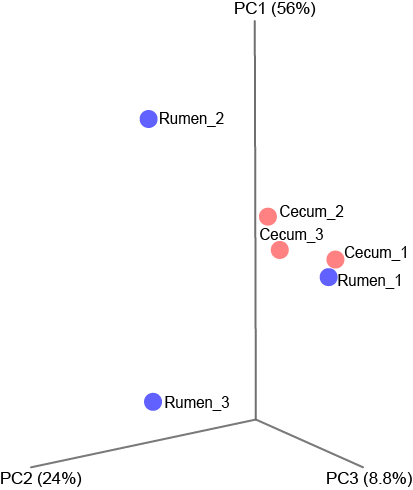

Supplement: Figure S2 — 16S rRNA gene surveys reveal hierarchical partitioning of all 6 samples. Methanogens communities were clustered using principal coordinate analysis of the full-tree-based Unifrac matrix. Each point corresponds to a sample colored to indicate locations in the GIT. Three principal components (PC1, PC2, and PC3) explained 88.8% variation. (TIF) [file pone.0114513.s002.tif]
