# Supplementary material for: Bacteria and Methanogens Differ along the Gastrointestinal Tract of Chinese Roe Deer (Capreolus pygargus)
Source: PLoS One. 2014 Dec 9;9(12):e114513. doi: 10.1371/journal.pone.0114513 (PMC4260832; doi:10.1371/journal.pone.0114513)
Supplement: Table S1 — The results of Basic Local Alignment Search Tool for the representative sequences of methanogen OTUs. OTU, operational taxonomic units. (DOCX) [file pone.0114513.s003.docx]

Table S1 The results of BLAST for the representative methanogens OTU sequences.

| OTUs | Nearest valid taxon | Max Score | Coverage | E Value | Seq^a^% |
| --- | --- | --- | --- | --- | --- |
| 1 | Methanobrevibacter millerae strain ZA-10 | 784 | 100 | 0 | 99 |
| 2 | Methanobrevibacter boviskoreani JH1 | 789 | 100 | 0 | 99 |
| 3 | Candidatus Methanomethylophilus alvus Mx1201 | 749 | 100 | 0.00E+00 | 98 |
| 4 | Methanobrevibacter woesei strain CH389 | 723 | 100 | 0 | 97 |
| 5 | Methanosphaera stadtmanae strain DSM 3091 | 721 | 94 | 0 | 99 |
| 6 | Methanobrevibacter millerae strain ZA-10 | 695 | 100 | 0 | 96 |
| 7 | Methanobrevibacter millerae strain ZA-10 | 734 | 100 | 0 | 97 |
| 8 | Methanobrevibacter ruminantium strain M1 | 750 | 100 | 0 | 98 |
| 9 | Methanosphaera stadtmanae strain DSM 3091 | 695 | 100 | 0 | 96 |
| 10 | Methanobrevibacter millerae strain ZA-10 | 773 | 100 | 0 | 99 |
| 11 | Candidatus Methanomethylophilus alvus Mx1201 | 638 | 100 | 2.00E-179 | 93 |
| 12 | Methanosphaera cuniculi strain 1R7 | 721 | 99 | 0 | 97 |
| 13 | Methanobrevibacter millerae strain ZA-10 | 726 | 100 | 0 | 97 |
| 14 | Methanobrevibacter millerae strain ZA-10 | 734 | 100 | 0 | 97 |
| 15 | Methanobrevibacter boviskoreani JH1 | 701 | 100 | 0 | 96 |
| 16 | Methanobrevibacter millerae strain ZA-10 | 723 | 100 | 0 | 97 |
| 17 | Methanobrevibacter smithii strain PS | 717 | 100 | 0 | 97 |
| 18 | Methanobrevibacter ruminantium strain M1 | 712 | 100 | 0 | 97 |
| 19 | Methanobrevibacter woesei strain CH389 | 667 | 100 | 0 | 95 |
| 20 | Methanobrevibacter boviskoreani JH1 | 717 | 100 | 0 | 97 |
| 21 | Methanobrevibacter millerae strain ZA-10 | 750 | 100 | 0 | 98 |
| 22 | Methanosphaera cuniculi strain 1R7 | 741 | 99 | 0 | 98 |
| 23 | Methanosphaera stadtmanae strain DSM 3091 | 767 | 100 | 0 | 99 |
| 24 | Methanobrevibacter millerae strain ZA-10 | 723 | 100 | 0 | 97 |
| 25 | Methanosphaera stadtmanae strain DSM 3091 | 695 | 100 | 0 | 96 |
| 26 | Methanobrevibacter millerae strain ZA-10 | 706 | 100 | 0 | 96 |
| 27 | Methanobrevibacter smithii strain PS | 673 | 100 | 0 | 95 |
| 28 | Methanobrevibacter millerae strain ZA-10 | 723 | 100 | 0 | 97 |
| 29 | Methanosphaera cuniculi strain 1R7 | 721 | 99 | 0 | 97 |
| 30 | Methanobrevibacter millerae strain ZA-10 | 712 | 100 | 0 | 97 |
| 31 | Methanosphaera stadtmanae strain DSM 3091 | 734 | 100 | 0 | 97 |
| 32 | Methanobrevibacter millerae strain ZA-10 | 723 | 100 | 0 | 97 |
| 33 | Methanobrevibacter boviskoreani JH1 | 717 | 100 | 0 | 97 |
| 34 | Methanosphaera cuniculi strain 1R7 | 710 | 99 | 0 | 97 |
| 35 | Methanosphaera stadtmanae strain DSM 3091 | 723 | 100 | 0 | 97 |
| 36 | Methanosphaera stadtmanae strain DSM 3091 | 667 | 100 | 0 | 95 |
| 37 | Methanobrevibacter millerae strain ZA-10 | 717 | 100 | 0 | 97 |
| 38 | Methanobrevibacter millerae strain ZA-10 | 723 | 100 | 0 | 97 |
| 39 | Methanosphaera stadtmanae strain DSM 3091 | 712 | 100 | 0 | 97 |
| 40 | Methanosphaera stadtmanae strain DSM 3091 | 728 | 100 | 0 | 97 |
| 41 | Methanosphaera cuniculi strain 1R7 | 688 | 99 | 0 | 96 |
| 42 | Methanosphaera cuniculi strain 1R7 | 715 | 99 | 0 | 97 |
| 43 | Methanosphaera cuniculi strain 1R7 | 688 | 99 | 0 | 96 |
| 44 | Methanobrevibacter olleyae strain KM1H5-1P | 750 | 100 | 0 | 98 |
| 45 | Methanobrevibacter millerae strain ZA-10 | 767 | 100 | 0 | 99 |
| 46 | Methanobrevibacter millerae strain ZA-10 | 717 | 100 | 0 | 97 |
| 47 | Methanobrevibacter millerae strain ZA-10 | 734 | 100 | 0 | 97 |
| 48 | Methanobrevibacter millerae strain ZA-10 | 728 | 100 | 0 | 97 |
| 49 | Methanobrevibacter millerae strain ZA-10 | 756 | 100 | 0 | 98 |
| 50 | Methanosphaera cuniculi strain 1R7 | 676 | 99 | 0 | 95 |
| 51 | Methanobrevibacter millerae strain ZA-10 | 689 | 100 | 0 | 96 |
| 52 | Methanobrevibacter millerae strain ZA-10 | 761 | 100 | 0 | 99 |
| 53 | Methanosphaera stadtmanae strain DSM 3091 | 695 | 100 | 0 | 96 |
| 54 | Methanosphaera cuniculi strain 1R7 | 682 | 99 | 0 | 95 |
| 55 | Methanobrevibacter millerae strain ZA-10 | 728 | 100 | 0 | 98 |
| 56 | Methanobrevibacter olleyae strain KM1H5-1P | 750 | 100 | 0 | 98 |
| 57 | Methanobrevibacter millerae strain ZA-10 | 736 | 100 | 0 | 97 |
| 58 | Methanobrevibacter millerae strain ZA-10 | 767 | 100 | 0 | 99 |
| 59 | Methanobrevibacter millerae strain ZA-10 | 695 | 100 | 0 | 96 |
| 60 | Methanosphaera stadtmanae strain DSM 3091 | 695 | 100 | 0 | 96 |
| 61 | Methanobrevibacter ruminantium strain M1 | 712 | 100 | 0 | 97 |
| 62 | Methanosphaera stadtmanae strain DSM 3091 | 761 | 100 | 0 | 97 |
| 63 | Methanosphaera stadtmanae strain DSM 3091 | 695 | 100 | 0 | 96 |
| 64 | Methanobrevibacter ruminantium strain M1 | 712 | 100 | 0 | 97 |
| 65 | Methanocorpusculum labreanum strain Z | 739 | 100 | 0 | 98 |
| 66 | Methanosphaera stadtmanae strain DSM 3091 | 706 | 100 | 0 | 96 |
| 67 | Methanosphaera stadtmanae strain DSM 3091 | 745 | 100 | 0 | 97 |
| 68 | Methanobrevibacter olleyae strain KM1H5-1P | 712 | 100 | 0 | 97 |
| 69 | Methanobrevibacter ruminantium strain M1 | 734 | 100 | 0 | 97 |
| 70 | Methanobrevibacter millerae strain ZA-10 | 712 | 100 | 0 | 97 |
| 71 | Methanobrevibacter millerae strain ZA-10 | 701 | 100 | 0 | 96 |
| 72 | Methanobrevibacter millerae strain ZA-10 | 728 | 100 | 0 | 97 |
| 73 | Methanosphaera stadtmanae strain DSM 3091 | 734 | 100 | 0 | 97 |
| 74 | Methanosphaera stadtmanae strain DSM 3091 | 723 | 100 | 0 | 97 |
| 75 | Methanosphaera cuniculi strain 1R7 | 710 | 99 | 0 | 97 |
| 76 | Methanobrevibacter boviskoreani JH1 | 695 | 100 | 0 | 96 |
| 77 | Methanobrevibacter arboriphilus strain AZ | 684 | 100 | 0 | 95 |
| 78 | Methanobrevibacter smithii strain PS | 728 | 100 | 0 | 97 |
| 79 | Methanobrevibacter millerae strain ZA-10 | 723 | 100 | 0 | 97 |
| 80 | Methanosphaera cuniculi strain 1R7 | 737 | 99 | 0 | 98 |
| 81 | Methanobacterium flexile strain GH | 695 | 100 | 0 | 96 |
| 82 | Methanosphaera cuniculi strain 1R7 | 710 | 99 | 0 | 97 |
| 83 | Methanosphaera cuniculi strain 1R7 | 715 | 99 | 0 | 97 |
| 84 | Methanobrevibacter millerae strain ZA-10 | 774 | 98 | 0 | 98 |
| 85 | Methanosphaera stadtmanae strain DSM 3091 | 737 | 99 | 0 | 97 |
| 86 | Methanobrevibacter millerae strain ZA-10 | 767 | 100 | 0 | 99 |
| 87 | Methanobrevibacter millerae strain ZA-10 | 695 | 100 | 0 | 96 |
| 88 | Methanobrevibacter millerae strain ZA-10 | 723 | 100 | 0 | 97 |
| 89 | Methanosphaera stadtmanae strain DSM 3091 | 717 | 100 | 0 | 97 |
| 90 | Methanosphaera cuniculi strain 1R7 | 715 | 99 | 0 | 97 |
| 91 | Methanobrevibacter millerae strain ZA-10 | 712 | 100 | 0 | 97 |
| 92 | Methanobrevibacter olleyae strain KM1H5-1P | 712 | 100 | 0 | 97 |
| 93 | Methanosphaera cuniculi strain 1R7 | 699 | 99 | 0 | 96 |
| 94 | Methanobrevibacter boviskoreani JH1 | 728 | 100 | 0 | 97 |
| 95 | Methanobrevibacter millerae strain ZA-10 | 750 | 100 | 0 | 98 |
| 96 | Methanosphaera cuniculi strain 1R7 | 710 | 99 | 0 | 97 |
| 97 | Methanobrevibacter millerae strain ZA-10 | 712 | 100 | 0 | 97 |
| 98 | Methanobrevibacter millerae strain ZA-10 | 723 | 100 | 0 | 97 |
| 99 | Methanosphaera stadtmanae strain DSM 3091 | 706 | 100 | 0 | 96 |
| 100 | Methanobrevibacter millerae strain ZA-10 | 765 | 100 | 0 | 99 |
| 101 | Methanobrevibacter thaueri strain CW | 734 | 100 | 0 | 97 |
| 102 | Methanobrevibacter millerae strain ZA-10 | 728 | 100 | 0 | 97 |
| 103 | Methanosphaera stadtmanae strain DSM 3091 | 739 | 100 | 0 | 98 |
| 104 | Methanobrevibacter smithii strain PS | 739 | 100 | 0 | 98 |
| 105 | Methanosphaera stadtmanae strain DSM 3091 | 689 | 100 | 0 | 96 |
| 106 | Methanobrevibacter millerae strain ZA-10 | 734 | 100 | 0 | 99 |
| 107 | Methanosphaera stadtmanae strain DSM 3091 | 739 | 100 | 0 | 98 |
| 108 | Methanobrevibacter millerae strain ZA-10 | 723 | 100 | 0 | 97 |
| 109 | Methanobrevibacter millerae strain ZA-10 | 706 | 100 | 0 | 96 |
| 110 | Methanobrevibacter boviskoreani JH1 | 717 | 100 | 0 | 97 |
| 111 | Methanobrevibacter boviskoreani JH1 | 717 | 100 | 0 | 97 |
| 112 | Methanobrevibacter boviskoreani JH1 | 701 | 100 | 0 | 96 |
| 113 | Methanobrevibacter boviskoreani JH1 | 723 | 100 | 0 | 97 |

a Percentage sequence identity to valid taxon
